# Supplementary material for: Identification of photoperiod-induced specific miRNAs in the adrenal glands of Sunite sheep (Ovis aries)
Source: Front Vet Sci. 2022 Jul 22;9:888207. doi: 10.3389/fvets.2022.888207 (PMC9354845; doi:10.3389/fvets.2022.888207)
Supplement: Supplementary file 1 [file Data_Sheet_1.ZIP › Supplementary file/miRDeep2 file.zip!/miRDeep2 file/Novel_Structure/SP42A3/Novel_112.pdf]

Provisional ID	:	NC_019482.2_30716
Score total	:	1.7
Score for star read(s)	:	-1.3
Score for read counts	:	0
Score for mfe	:	1.4
Score for randfold	:	1.6
Score for cons. seed	:	
Total read count	:	103
Mature read count	:	103
Loop read count	:	0
Star read count	:	0

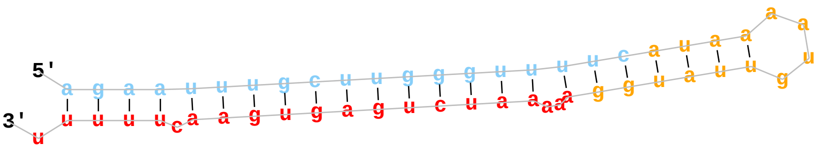

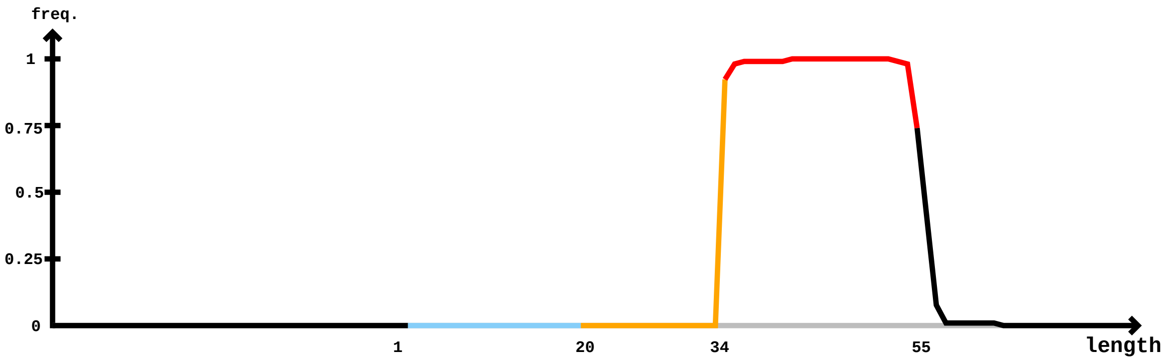

Star Mature

```
5'- accauuauauacagaugauuuagugaguuggccaaagaauuugcuuggguuuucauaaaauguuauggaaaaaucugagugaacuuuuuggccaacggaauauuuuucucc   -3'    exp
    ..(((((.............))))).((((((((((((.(((((((((((((((((((....)))))))..))))))))))))).))))))))))))((((.....))))..    reads        mm          sample
    ....................................................................aaaaaucugaAugaacuu.........................    1            1           seq
    ....................................................................aaaaaucugagugaacuuu........................    1            0           seq
    ....................................................................aaaaaucugaAugaacuuuu.......................    24           1           seq
    ....................................................................aaaaaucugagugaacuuuu.......................    1            0           seq
    ....................................................................aaaaaucugaAugaacuuuuu......................    69           1           seq
    .....................................................................aaaaucugagugaacuuuuugg....................    6            0           seq
    ......................................................................aaaucugagugaacuuuuugg....................    1            0           seq
    ............................................................................ugagugaacuuuuuggccaacU.............    1            1           seq
```
